# P-1524. Clinical Characteristics and Outcomes of Patients with *Candida auris* at RUHS

**DOI:** 10.1093/ofid/ofae631.1693

**Published:** 2025-01-29

**Authors:** Chiao An Chiu, Maily Hong, Arunmozhi Aravagiri-Do, Rebecca Nguyen, Nikki Mulligan, Made Sutjita, Bruce Weng

**Affiliations:** Riverside University Health System Medical Center, Moreno Valley, California; Thomas J. Long School of Pharmacy, Stockton, California; Riverside University Health System, Moreno Valley, California; Riverside University Health System, Moreno Valley, California; Riverside University Health System Medical Center, Moreno Valley, California; Riverside University Health System, Moreno Valley, California; Riverside University Health System Medical Center, Moreno Valley, California

## Abstract

**Background:**

- *Candida auris* is an emerging yeast that poses significant public health risks due to its resistance to multiple classes of antifungal medications.
- Outbreaks in hospitals and long-term care facilities are often characterized with high mortality and morbidity. Based on California Department of Public Health surveillance, Riverside county ranked top 4 in reported *C. auris* cases.
- We aim to describe the clinical characteristics, clinical associations, and antifungal susceptibility pattern and outcomes of *C. auris* blood stream infection at Riverside University Health System (RUHS).

Invasive Candidiasis Clinical Associations

**Figure:**
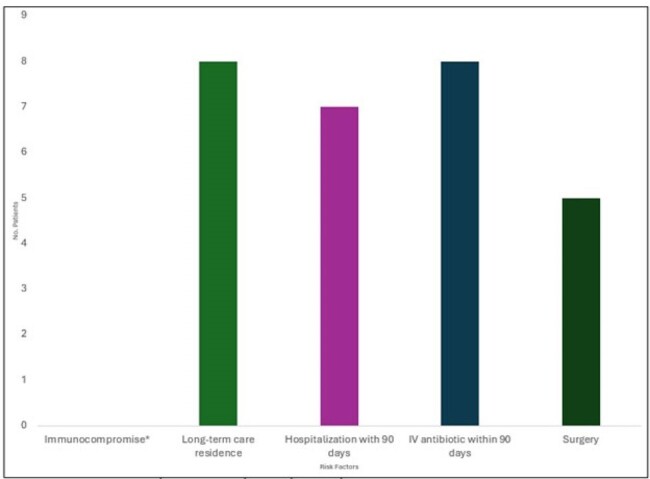

*: Immunocompromised status including patients on chemotherapy, status post organ transplant, HIV positive, etc.

**Methods:**

We conducted a retrospective chart review of *C. auris* fungemia cases in adult hospitalized patients at RUHS between January 2022 to December 2023. Demographic data, clinical associations, antifungal susceptibility and clinical outcomes were noted using descriptive statistics.

Antifungal Susceptibility

**Figure:**
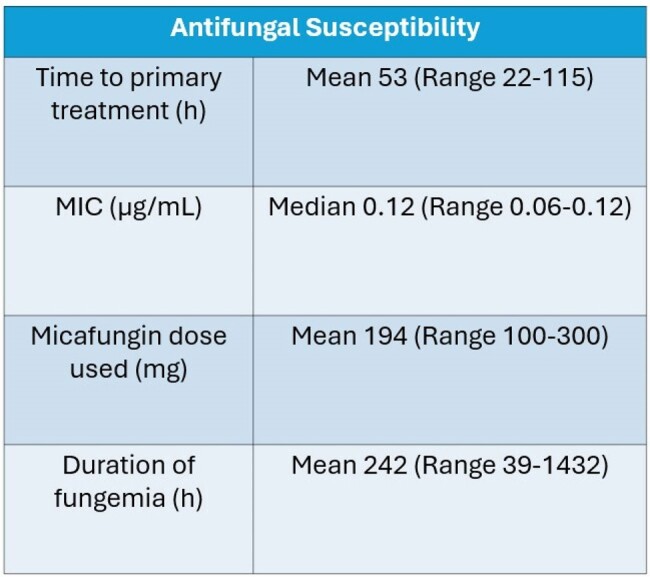

**Results:**

- Nine patients (n = 9) were identified. 8/9 (89%) presented from a long-term care facility. 5/9 (56%) patients were male gender averaging 65 years old (range 45-86yo). Charlson comorbidity index was 8.11.
- Clinical associations: invasive devices (9/9, 100%), long-term care residence (8/9, 89%), hospitalization within 90 days (7/9, 78%), IV antibiotic within 90 days (8/9, 89%), and surgery (5/9, 56%).
- All patients were treated with micafungin. Despite 100% susceptibility to echinocandins, fungemia duration was long (mean 242.1 hours; range 39.13-1431.75 hours) and 4/9 (44%) patients required a dose increase of micafungin (mean dose 194 mg; range 100-300 mg).
- Cases were further characterized by prolonged ICU length of stay (LOS (mean 15d, range 3-41d)), prolonged hospital LOS (mean 39.3d, range 9-65d), and high mortality (4/9, 44%).
- 2/5 (40%) survivors versus 0/4 (0%) deceased patients had source control. The majority of survivors 4/5 (80%) had a primary line-associated fungemia. 2/5 survivors (40%) were readmitted within 6 months.

Mortality in Relation to Source Control

**Figure:**
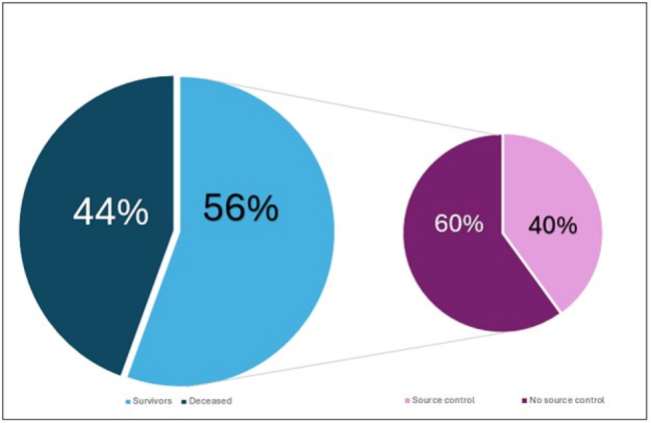

**Conclusion:**

- Patients from long-term care facility with additional clinical associations appear to have high risk for *C. auris* infection and complications.
- Consideration of higher initial dose echinocandins should be considered in management of patients presenting with *C.auris* fungemia.
- Aggressive source control of fungemia etiology should be pursued when possible.

**Disclosures:**

**All Authors**: No reported disclosures

